# Efficacy of in-service nutrition training for mid-level providers to improve feeding practices among HIV-positive children in Tanga, Tanzania: study protocol for a cluster randomized controlled trial

**DOI:** 10.1186/1745-6215-14-352

**Published:** 2013-10-25

**Authors:** Bruno F Sunguya, Krishna C Poudel, Linda B Mlunde, David P Urassa, Masamine Jimba, Junko Yasuoka

**Affiliations:** 1 Department of Community and Global Health, Graduate School of Medicine, The University of Tokyo, 7-3-1, Hongo, Bunkyo-ku, Tokyo 113-0033, Japan; 2 Department of Public Health, School of Public Health and Health Sciences, University of Massachusetts Amherst, Arnold House, 715 North Pleasant St, Amherst, MA 01003-9304, USA; 3 School of Public Health and Social Sciences, Muhimbili University of Health and Allied Sciences, P.O. Box 65489, Dar es Salaam, Tanzania

**Keywords:** Children, HIV/AIDS, Feeding practices, Mid-level providers, Nutrition status, Undernutrition

## Abstract

**Background:**

Feeding practices and child undernutrition can be improved when trained health workers provide proper nutrition counseling to caregivers. However, this important management component is difficult to achieve in countries where trained health workers are limited; Tanzania is no exception. In rural and semi-urban areas, mid-level providers (MLPs) are left to manage diseases such as HIV/AIDS.

Training health workers in nutrition has been shown to be an effective intervention among HIV-negative children elsewhere, but no studies have been conducted among HIV-positive children. Furthermore, in Tanzania and other countries with MLPs, no evidence currently exists demonstrating an improvement in nutrition among children who receive health services given by MLPs. This study thus aims to examine the efficacy of nutrition training of MLPs on feeding practices and the nutrition status of HIV-positive children in Tanga, Tanzania.

**Methods/Design:**

We will conduct a cluster randomized controlled trial in care and treatment centers (CTCs) in Tanga, Tanzania. The CTCs will be the unit of randomization. We will select 16 CTCs out of 32 for this study, of which we will randomly assign 8 to the intervention arm and 8 to the control arm by coin flipping. From the selected CTCs we will attempt to recruit a total of 800 HIV-positive children aged 6 months to 14 years, half of whom will be receiving care and/or treatment in the CTCs of the intervention arm, and the other half of whom will be receiving care and/or treatment in the CTCs of the control arm (400 children in each condition).

We will provide nutrition training to MLPs of the CTCs selected for the intervention arm. In this intervention, we will use the World Health Organization guidelines on nutrition training of health workers for HIV-positive children aged 6 months to 14 years. The trained MLPs will then provide tailored nutrition counseling to caregivers of children being treated at the 8 CTCs of the intervention arm. We will measure nutrition status and child feeding practices monthly for a total of six months.

**Conclusions:**

Results of this trial will help expanding undernutrition interventions among HIV-positive children in Tanzania and other countries.

**Trial registration:**

Current Controlled Trials: ISRCTN65346364.

## Background

HIV/AIDS has exacerbated childhood undernutrition in developing countries and has led to a high mortality rate [1]; Tanzania is no exception. Among the general population of Tanzania, about 5.7% (2 million) were estimated to be living with HIV/AIDS in 2009. Meanwhile, 47.8% of children in the general population suffer from stunting or a chronic form of undernutrition [2]. Undernutrition among HIV-positive children remains a serious problem, even when antiretroviral therapy (ART) is administered [3]. In Tanzania, ART-treated, HIV-positive children were more likely to suffer from being underweight (4.6 times) and from wasting (9.6 times) compared to HIV-negative children [3].

Among HIV-positive children, undernutrition can be controlled if well-trained health workers counsel caregivers frequently [4,5]. By knowing the determinants of undernutrition in the specific population, such nutrition counseling can be carried out much more easily. The counseling may also focus on feeding behaviors and practices using locally available foods. Nutrition monitoring and early treatment of undernutrition also prevent further deterioration among children with pre-existing undernutrition. However, as the number of trained health workers in Tanzania is limited [6], such children have continued to suffer an unacceptable toll of undernutrition [3,7]. Semi-urban and rural areas bear the biggest brunt, and while mid-level providers (MLPs) are based in these areas [8], they are often not trained enough to ensure appropriate nutritional support of these children.

MLPs usually treat simple and specific diseases and perform simple surgical procedures [9]. They are health workers with 2–3 years of post-secondary school healthcare training who undertake tasks usually carried out by doctors and nurses [9]. The MLPs’ training focuses on major ailments specific to the populations they serve. In Tanzania, like in many other developing countries, such health cadres are commonly found in small cities and semi-urban and rural areas. In the 1960s, Tanzania introduced MLP cadres to solve the health workforce crisis in the country. Their training, however, lacks basic scientific components such as nutrition science as well as skills such as counseling, both of which are vital in helping people to improve their feeding practices and management of undernutrition. As a result, MLPs do not always have adequate knowledge and skills to manage child undernutrition, especially when it is complicated by HIV/AIDS.

In-service nutritional training of qualified health workers, such as medical doctors, nurses, nurse-midwives, nutritionists, and dieticians, can improve feeding practices including feeding frequency, dietary diversity, and energy intake among HIV-negative children aged 6 months to 2 years [10]. Nutrition counseling has been shown to have a positive effect on complementary feeding and nutrition status of children when it is provided by health workers who themselves received in-service nutrition training [11-13]. However, the evidence for such positive effects is only available for HIV-negative populations managed by well-trained health workers such as doctors and nurses—not MLPs. Furthermore, even in the case of well-trained health workers, the evidence base for the effectiveness of such interventions in sub-Saharan Africa is lacking. It is therefore difficult to generalize the results of the effectiveness of in-service nutrition training of qualified health workers to the HIV-positive children and health cadres below the qualified medical personnel in sub-Saharan Africa. This study’s main objectives are to examine the efficacy of nutrition training of MLPs on improving feeding practices and the nutrition status of HIV-positive children, as well as to improve the MLPs nutrition knowledge, management, and counseling skills in relation to child undernutrition, in Tanga, Tanzania.

## Methods/Design

### Study area

We will conduct this study in HIV Care and Treatment Centers (CTCs) in Tanga, in the North Eastern region of Tanzania. Tanga is a coastal region with varied climatic conditions that allow diverse food production. Even if sisal market exports are lost, Tanga remains one of the main producers of cereals and fruits, and these make up a large part of its major economic activities.

Tanga is a food secured region; however, it has an unacceptably high proportion of child undernutrition. For example, in the nationally representative survey in 2010, more than 49% of the 315 under-five children surveyed in the Tanga region were stunted [2]. Acute undernutrition among them is also rampant; about 12% of the 315 under-fives were underweight while 5.5% were suffering from wasting. The regional diversity in food production does not reflect diversity in consumption. For example, Tanga produces various cereals, fruits, vegetables, and both fresh and sea water products; however, only 59.4% of the 292 children aged 6 to 60 months sampled from the general population consumed foods rich in vitamin A [14]. The proportion of vitamin A deficiency among these children was 38.9%, iron deficiency was 36.5%, and iron deficiency anemia was 52.2% [14].

The region is also not spared by HIV/AIDS epidemic. The prevalence of HIV/AIDS in the region is estimated to be 2.4%, based on a sample of 833 adults aged 15 to 49 years old who were tested as part of a nationally representative survey [15,16]. Data of nutrition profiles of HIV-positive children in this region is not available.

CTCs are specialized clinics to care for HIV-positive people, including children. They operate under vertical HIV programs and are integrated into the existing health infrastructure [17]. In Tanga, all CTCs are located in health facilities. They provide care and treatment to HIV-positive people including voluntary counseling and HIV testing, prescribing and dispensing ART, ART adherence counseling, diagnosis and treatment of opportunistic infections, monitoring response to treatment and disease progression, behavior change promotion, and outreach services, among others [15,17]. HIV-positive people attend CTCs every month to get ART refills. In each visit, they also receive adherence counseling and monitoring, examination by clinicians, and management of opportunistic infections, if any [15,17].

Until December 2012, the Tanga region had a total of 32 CTCs. They are distributed throughout the region based on population density and the needs of specific areas. All districts have CTCs located in the district hospital and health centers. A total of 20,773 people living with HIV/AIDS were enrolled at these CTCs by the year 2009 [15]. According to regional unpublished data, about 1,800 HIV-positive children were enrolled at CTCs for care and treatment as of March 2013.

### Study design

**Figure 1:**
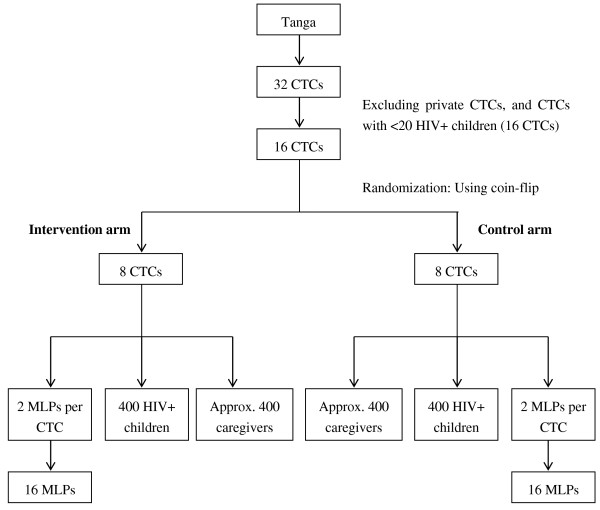
Participants selection flow chart.

We will conduct a cluster randomized controlled trial in Tanga, Tanzania. We will select 16 CTCs out of 32 for this study (Figure 1). The selected CTCs are located in public health facilities and have at least 20 enrolled HIV-positive children. We will use the coin-flipping method to randomly assign 8 CTCs to the intervention arm and 8 to the control arm (Figure 1). We will examine the effectiveness of nutrition training of MLPs on their nutrition knowledge, management of undernutrition, feeding practices, and the nutrition status of HIV-positive children attending CTCs. The intervention follows formative research conducted using focus group discussions and a cross-sectional study to examine factors associated with undernutrition, available foods, and local feeding practices for the children in this population. Results of the formative research are in the process of being published in another journal.

#### Baseline

We will assess nutrition status and feeding practices (feeding frequency, dietary diversity, quality and quantity of food items consumed in the previous 24 hours) of the recruited children. We will investigate the burden of disease caused by opportunistic infections, diarrhea, malaria, and intestinal helminthes. We will also assess MLPs’ nutrition knowledge and skills for managing undernutrition in children living with HIV/AIDS before and after the training.

#### Intervention

We will conduct in-service nutrition training of two MLPs selected from each of the CTCs in the intervention arm, providing training on how to prevent, diagnose, and manage undernutrition based on the identified local determinants of undernutrition. The training methods will be adopted from the standard *Integrated Management of Childhood Illness*: *Counsel the Mother* handbook [18] and the *Guidelines for an Integrated Approach to the Nutritional Care of HIV*-*infected Children* (*6 months to 14 years*) [19].

At the end of the nutrition training, health workers are expected to improve in two competencies: knowledge on nutrition and feeding practices and skills on managing and providing nutrition care for HIV-positive children [19]. We will provide pre-course materials that include information on HIV/AIDS-staging, basic counseling skills, and basic information on complementary feeding of children to the MLPs.

The course structure includes 18 sessions of lectures, demonstrations, practice sessions, exercises, and role-play. According to the standard WHO training guide [19], this training will take 13 hours and 40 minutes. We will conduct this training over the course of two consecutive days. Table 1 contains the estimated duration and description of each session.

**Table 1:** Nutrition training content and session duration

**Session**	**Content**	**Session duration**
Session 1	Introduction	15 minutes
Session 2	Review of pre-course materials	60 minutes
Session 3	Assess growth using growth charts	40 minutes
Session 4	Measuring growth using growth charts	30 minutes
Session 5	Listening and learning counseling skills	30 minutes
Session 6	Clinical signs of malnutrition in a child	30 minutes
Session 7	Practical session 1: Assessing and classifying a child’s weight and growth	120 minutes
Session 8	Assess child’s nutrition needs	30 minutes
Session 9	Decide on a Nutritional Care Plan	60 minutes
Session 10	What does the child eat or drink?	45 minutes
Session 11	Who feeds the child and how does the child eat?	30 minutes
Session 12	Food security	30 minutes
Session 13	Building confidence and support skills	30 minutes
Session 14	Practical session 2: Assessing and classifying a child’s nutritional status	120 minutes
Session 15	Exercise, risk factors and referral	30 minutes
Session 16	The HIV-infected child with special needs	60 minutes
Session 17	Children on antiretroviral treatment	30 minutes
Session 18	Conclusion and competencies	30 minutes

Total training duration 13 hours 40 minutes.

According to the requirements set by the training guide, we will select trainers who are experienced in teaching the IMCI guide, managing undernutrition and HIV/AIDS among children, counseling, and recommending feeding practices for children [19]. We will include one pediatrician, one nutritionist, and one psychologist in the team of trainers. They will be provided with the trainer’s guide and the participants’ guide, as well as the booklet that comes along with the other training materials [19].

The trained MLPs will provide nutrition counseling to caregivers and will separately undertake direct measures for managing undernutrition to the HIV-positive children attending the CTCs of the intervention arm on a monthly basis for six months of the follow-up period. A similar intervention will be made available for the control group after evaluating the results.

#### Follow-up

We will follow-up both intervention and control groups for 6 months. We will evaluate feeding practices, such as feeding frequency, dietary diversity, and quality and quantity of food eaten by children, as well as nutrition status, on a monthly basis for six months. For the intervention arm, we will also measure nutrition knowledge of MLPs before the training, immediately after the nutrition training, and again after the six-month observation period in order to measure the degree of knowledge decay.

### Participants and selection criteria

Participants of this study will include MLPs who manage HIV-positive children attending the CTCs in Tanga and pairs of HIV-positive children and their caregivers.

#### Midlevel providers (MLPs)

We will invite a minimum of two MLPs from each CTC of the intervention arm to take part in the nutrition training. We will also recruit a similar number of MLPs from the CTCs of the control arm. A total of 32 MLPs will participate in this study. MLPs include assistant medical officers, clinical officers, allied health workers, and nurse assistants. We will exclude all other health workers that do not fulfill the MLP criteria as per its definition. Such health workers include health promotion volunteers and non-clinician, home-based care staff.

#### HIV-positive children

We will recruit HIV-positive children attending the selected CTCs. Inclusion criteria for participation of the HIV-positive children are: aged 6 months to 14 years; registered or transferred to the CTCs; attended with his/her caregiver; consent by the caregiver to voluntarily participate in the study. We will exclude children whose ART information is missing in the medical records and whose HIV sero-status has not been confirmed. An estimated 800 children currently attend the 16 selected CTCs. Because half of the CTCs will be selected for the intervention arm, approximately half of the potential participants (400) will be in the intervention group and a similar number for the control group.

#### Caregivers of the HIV-positive children

We will recruit caregivers of HIV-positive children for this study because our study involves children aged 6 months to 14 years, who cannot give consent themselves to participate in the study nor can they participate in the study alone. Furthermore, the nutrition counseling conducted by the MLPs will target the children’s caregivers. We will therefore be recruiting a similar number of caregivers as there will be children. As in previous studies [3,20], we will use the definition of a caregiver as a child’s parent, relative, guardian, or anyone else above 18 years old who takes care of the child, supervises their treatment, and accompanies the child to the CTC.

#### Sample size estimation

To calculate the minimum sample size for the intervention and control group, we assumed the inter-cluster coefficient to be as low as 0.01. Also, we assumed that 8 clusters (CTCs) would be included in the intervention arm and a similar number in the control arm. We could not find any other studies, based in sub-Saharan Africa, that estimated weight gain after implementing this type of intervention, and therefore we used a similar study conducted in China investigating children in the general population as a reference [21]. In this study, an estimated mean weight gain difference between children of the intervention and control arm was 0.3 kg in six months. To detect the mean difference of 0.3 kg weight gained in the intervention group compared to the control group over a 6-month duration, at a power of 80% and 5% significant level, 24 participants per cluster will be needed. In total, a minimum sample size of 192 participants for each arm will be required to produce the desired effect. To counteract the effect of loss to follow-up, deaths, a number of outcome variables, and missing data, we will attempt to recruit 400 HIV-positive children for the intervention arm and a similar number for the control arm. Therefore, an estimated 800 caregivers will also be recruited to participate in this study, with a similar distribution between the intervention and control groups (400 in each arm).

### Randomization process

We will use the CTCs as the unit of randomization. A total of 32 CTCs provide care and treatment to about 1,800 HIV-positive children aged 0 to 14 years old in Tanga. We will exclude private CTCs, as well as CTCs with less than 20 HIV-positive children. A total of 16 CTCs in the area are public and have at least 20 HIV-positive children receiving care and/or treatment; they also represent all the districts of the region. Of the 16 CTCs, 4 are from the Tanga urban area; this is due to a high number of CTCs and HIV-positive patients attending them. All CTCs based in district hospitals will be selected in this study because they fulfill the selection criteria. These 16 CTCs will be eligible for the randomization process: a coin toss to assign 8 CTCs to the intervention arm and 8 to the control arm. A person who is not a member of the study research team will conduct the randomization process.

### Measurements

#### Nutrition status

We chose the nutrition status of HIV-positive children as the outcome variable, including use of the categories underweight, wasting, and stunting. We will measure children’s weight using a standardized hanging Salter® scale (UK) calibrated to 0.1 kg for children who cannot stand and a standardized Seka® digital scale (Brooklyn, USA) for children who can stand. Height will be measured for children aged 24 months and older using a Seka® measuring rod calibrated to 0.5 cm [22]. We will use a designated mark on a board to measure lengths of children younger than 24 months in a recumbent position [23].

After obtaining height and weight data, we will convert them to a height-for-age z-score, weight-for-age z-score, and weight-for-height z-score [24] using the Epi-Info ENA Ver. 3.5.1, 2008 (CDC, Atlanta, Georgia, USA) software and WHO reference values [25,26]. Low height-for-age, weight-for-height, and weight-for-age are measures of stunting, wasting, and underweight, respectively [27].

#### Dietary diversity

Dietary diversity is one of the measures of feeding practices and thus is one of our outcome variables. We will calculate a total dietary diversity score from a recalled list of food items consumed over the previous day. We will use a set list of 12 main food items found to be common in the formative research and the child questionnaire of the Tanzania Demographic and Health Survey [2,28]. We adopted this method as it has been used in previous studies among HIV-positive children in Tanzania [3,20].

#### Feeding frequency

Feeding frequency is also a measure of feeding practices and therefore one of our outcome variables. We will assess the feeding frequency of HIV-positive children by asking the caregivers to recall the number of times they fed their children in the 24 hours preceding the interview. WHO recommends feeding frequency of five times per day for HIV-positive children [19]. We will consider a feeding frequency of below five to be a low feeding frequency.

#### Quantity of food and quality of food

Amount and quality of food consumed by a child is again one of the measures of feeding practices and therefore an outcome variable in our study. We will measure food quality (ingredients or composition) and quantity using Tanzania food composition tables [29]. Such food composition tables provide information on calories, protein, vitamins, and essential minerals per 100 g of the local food type.

Adequate energy intake for HIV-positive children with no infections or severe undernutrition is 10% more than the requirement of normal children of the same age [19]. For HIV-positive children with opportunistic infections, energy requirements are 20% to 30% above normal children of the same age [19]. HIV-positive children with severe undernutrition have energy requirements of 50% to 100% above the required energy intake of HIV-negative children of the same age [19]. We will estimate the foods consumed in the previous 24 hours and work out specific ingredients. We will measure the amount consumed using common feeding utensils, such as plates, bowls or cups. We will categorize the diet as being low quantity if the child consumed an amount below the recommended value for that age.

#### Household food insecurity

We will measure household food insecurity using the Household Food Insecurity Access Scale (HFIAS) [30]. This is because food insecurity was associated with both acute and chronic forms of undernutrition among ART-treated HIV-positive children in Tanzania [3]. This scale has nine items on food access experience. Options are ranked from 0 = 'no’, 1 = 'rarely’, 2 = 'sometimes’, and 3 = 'often’. Score ranges from 0 to 27; higher scores reflect more severe food insecurity. The scale can continuously measure food insecurity, or it can be used categorically to identify food secure or insecure households. The recall duration is shorter compared to other scales; HFIAS uses a 30-day recalling period and has been used among the HIV-positive population in Kenya and Uganda [31-36].

### HIV CTC related data

#### HIV clinical stage

Advanced HIV clinical stage is associated with chronic undernutrition in Tanzania [3,20]. Like in the previous studies, we will use WHO clinical staging to determine HIV/AIDS progression. The four-stage classification uses both medical history and physical examination to classify HIV/AIDS progression. The first two clinical HIV stages are regarded as 'early stages’, whereas clinical HIV stages 3 and 4 are regarded as 'advanced stages’ [37]. In this study, we will extract the highest reached WHO clinical stage from the medical file of each child attending the CTC. The CTC staff updates such information on a routine basis with each CTC visit.

#### ART treatment duration

ART halts HIV/AIDS progression for HIV-positive individuals, improves their immunity, reduces opportunistic infections, and improves appetite [38-40]. Thus, it may also serve to ameliorate the worsening of nutrition status among HIV-positive children. ART treatment duration has also been associated with improvement of underweight and stunting [41]. In this study, we will measure the ART treatment duration as months since initiation.

We will check and record the ART regimen used. According to the national guidelines [17], HIV-positive children in Tanzania are typically treated by combination therapy of three ARTs, referred to as 'highly active antiretroviral therapy’. The fixed combination may either be two nucleoside reverse transcriptase inhibitors and a non-nucleoside reverse transcriptase inhibitor, or two nucleoside reverse transcriptase inhibitors and boosted protease inhibitor. Appropriate combinations differ with child age, anemia status, liver enzymes, and side effects. Children aged up to 36 months are given a combination therapy that includes Zidovudine, Lamivudine and Niverapine. Older children receive a combination of Zidovudine, Lamivudine and Efavirenz. If the child was exposed to Nevirapine on prevention of mother-to-child transmission of HIV/AIDS intervention during pregnancy, Niverapine is usually substituted with Lopinavir boosted with Ritonavir. Stavudine is used in place of Zidovudine if the child is diagnosed with anemia.

#### Opportunistic infections

We will also assess the presentation of any common opportunistic infections. Previous studies showed an association of opportunistic infections with undernutrition among children living with HIV/AIDS in Tanzania [3,42], and other countries in sub-Saharan Africa [40,43,44]. Such opportunistic infections include diarrhea, malaria, tuberculosis, upper respiratory tract infections, and oral/esophageal candidiasis. Diarrhea is defined as the presence of three or more watery stools during the previous 24 hours [45]. Malaria is defined as a typical febrile illness characterized by fever, chills and sweating, and evidenced by parasitological examination [46]. We will assess other opportunistic infections based on a medical history and medical records. We will also examine for intestinal helminthes among the children, as it is an important determinant of undernutrition, and in particular micronutrient deficits.

#### Socioeconomic position

We will assess economic status using a weighted wealth index. The index incorporates household durable assets ownership, such as owning a paraffin lamp, television, radio, telephone, flat iron, refrigerator, bicycle, motor car, farm and having electricity; housing and dwelling characteristics including main floor materials, house ownership, fuel for lighting and cooking, type of toilet, source of water, feeding characteristics, and household food satisfaction [2,28]. We will construct dichotomous variables for these items and carry out factor analysis using principle component analysis to reduce such variables into ones that will load as factor 1, which describe the socioeconomic position of the study population. We will use factor loadings as item weights, and sum them to yield the wealth index for each household [47-49]. We will divide the total weighted wealth index score into quartiles to designate levels of economic status.

#### Sociodemographic data

We will adopt other sociodemographic variables pertaining to children and their caregivers from the women and household questionnaires of the Tanzania Demographic and Health Survey [2,28]. Population surveys in Tanzania have already tested and used these variables both in 2005 and 2010. Such variables will include education level, orphanhood, religion, and marital status.

We will measure education level by years spent in school. We will also group religions into the common denominations found in Tanzania [50]. This includes Christians, Moslems, and non-religious. We will group marital status into either currently married or not currently married [2]. We will consider caregivers who are divorced or widowed at the time of data collection as 'not currently married’. We will measure child and caregiver’s ages in months and years, respectively [2,3].

### Data collection

Prior to data collection, we will train research assistants on the data collection and ethical procedures. We will translate the English questionnaire into Swahili and then back-translate it to English using two independent local researchers to check that is retains its original meaning. We will use the Swahili version of the questionnaire for data collection. The trained research assistants will conduct a pre-test for the questionnaire and the interview in the Swahili language. We will collect data from August 2013 and continue to do so monthly for a period of six months. To assess changes in the MLPs knowledge, we will use the questionnaire from the training manual to collect data before and after the nutrition training.

### Data analysis

We will analyze the data based on the intention-to-treat principle. We will use both descriptive and regression analyses. For the descriptive analyses, we will conduct both χ^2^ and Student’s *t*-tests to examine the differences in characteristics of participants in the intervention and control groups. This study will have two types of outcome variables. First, nutrition status measured as underweight, wasting, and stunting. Second, feeding practices measured by feeding frequency, dietary diversity, and the quality and quantity of foods consumed. We will use multiple logistic regression analyses to examine the effectiveness of nutrition training on nutrition status and feeding practices of the two arms while adjusting for confounding variables. We will also conduct analyses to see monthly changes in feeding practices and nutrition status and to compare the intervention and control arms. To examine the independent association of nutrition counseling by trained MLPs on outcome variables, we will use repeated measures of ANOVA and Generalized Estimated Equation.

### Ethical considerations

Before conducting interviews or allowing participation in the study, we will obtain written informed consent from the caregivers. Participants will be assured of the confidentiality and anonymity of reports and publications generated from this study. Participation will be voluntary and participants will be assured that there will be no implications for their care if they refuse to participate in the study. This study is approved by the Expedited Review Sub-Committee of the Senate Research and Publication Directorate of the Muhimbili University of Health and Allied Sciences in Tanzania and the Research Ethics Committee of the University of Tokyo in Japan.

## Discussion and implications

Nutrition training of health workers has been shown to be effective in improving the nutrition status of HIV-negative children. In our recent systematic review [10], health workers who were given in-service nutrition training could transform feeding practices and the energy intake of children in the general population. However, we could not find studies that targeted HIV-positive children. This population bears a higher burden of undernutrition compared to the general population. It is thus important to understand the effectiveness of such an intervention among this population.

Tanzania, along with other developing countries, also faces a shortage of well-trained health workers [6,51]. MLPs are left with the high burden of taking care of patients with minimum knowledge [8]. Our study will focus on the training of this health cadre and the examination of the efficacy of such training on management of undernutrition for HIV-positive children.

This study has the potential to directly transform the nutrition status and feeding practices of HIV-positive children in a region with a high burden of undernutrition. The findings of this study could support proposals to scale-up nutrition interventions using the available health infrastructure in semi-urban and rural areas of Tanzania, as well as in other countries with similar characteristics. We will also be able to better understand the local causes of undernutrition among HIV-positive children in an area that has adequate food production but a high toll of undernutrition.

The foreseen limitations of this study include differences in experiences and qualifications of targeted MLPs. Assistant medical officers have higher qualifications compared to clinical officers and assistant nurses. Their understanding of nutrition-related topics during the training might also be different. To minimize this limitation, we will use experienced trainers and both English and Swahili languages to emphasize the important points.

## Trial status

This trial was in the formulation phase during the preparation of this manuscript.

## Abbreviations

ART: Antiretroviral therapy; CTCs: Care and treatment centers; HFIAS: Household food insecurity access scale; MLPs: Mid-level providers; RCT: Randomized controlled trial.

## Competing interests

All authors declare that they have no competing interests.

## Authors’ contributions

BFS conceived the research questions, designed the study, and prepared the first draft, and will also supervise the intervention and conduct data collection and analyses. KCP refined the research questions and helped to prepare the first protocol. LBM contributed to the study design and helped to prepare the first draft. DPU revised the study design, prepared the protocol, and will supervise the interventions and data collection. JY participated in the preparation of the protocol, first draft and revisions. MJ reviewed the study protocol, supervised the trial, prepared the manuscript, and approved the submission. All authors read and approved the final version of the manuscript for submission.
